# Editorial: Drug-drug interactions in pharmacology

**DOI:** 10.3389/fphar.2023.1155738

**Published:** 2023-02-13

**Authors:** Simona Pichini, Annagiulia Di Trana, Oscar García-Algar, Francesco Paolo Busardò

**Affiliations:** ^1^ National Centre on Addiction and Doping, Istituto Superiore di Sanità, Rome, Italy; ^2^ Neonatology Unit, Hospital Clinic-Maternitat, ICGON, BCNatal, Barcelona, Spain; ^3^ Department of Excellence-Biomedical Sciences and Public Health, Università Politecnica Delle Marche, Ancona, Italy

**Keywords:** drug-drug interaction, pharmacology, pharmacokinetics, pharmacodinamics, ADME cycle

In pharmacology, drug-drug interactions result in unintended reactions, toxic side effects, or a lack of clinical efficacy in an individual body when multiple medications are simultaneously administered for one or more diseases (Molenaar-Kuijsten et al., 2021). These are usually considered in terms of two principal classes of underlying mechanisms: pharmacodynamics and pharmacokinetics (Nguyen et al., 2020). Indeed, the pharmacological effect of one or both drugs may be enhanced or suppressed, or a new and unanticipated adverse effect may occur, even leading to fatal consequences (Barbera et al., 2013; Karch et al., 2016).

Concerning pharmacokinetics, drug absorption, distribution, metabolism, and excretion (ADME cycle) variations may result in a plasma concentration fluctuation, influencing drug bioavailability. Interactions between drugs at the metabolic level can modify the metabolic enzymes altering drug activation or inactivation. If the metabolism is inhibited, it will remain longer in the body, so its concentration will increase, potentially causing secondary toxic effects. Conversely, metabolism enhancement can decrease plasma concentration and hence its bioavailability.

Pharmacodynamically, drugs can interact by binding to the same receptor. Two receptor agonists or two antagonists would increase the pharmacological actions of both, whereas an agonist and an antagonist would decrease each other’s pharmacological effects. In some interactions, drugs may produce biochemical changes that alter the sensitivity to toxicities produced by other drugs. Finally, whereas in the majority of cases, drug-drug interactions can cause toxic adverse effects (e.g., beta-blockers and bronchodilators; diuretics and steroids or digoxin; rifampicin and verapamil or carbamazepine), there are also several therapeutically beneficial drug interactions (e.g., docetaxel and piperine; resveratrol and diclofenac; ivermectin and lopinavir or saquinavir).

This Research Topic aims to provide original investigations, brief reports, and review papers concerning the latest insights into drug-drug interactions in pharmacology.

Recently, physiologically based pharmacokinetic (PBPK) models have been widely applied for the computational description of drug-drug interaction, since regulatory agencies have progressively accepted the model as predictive. To this concern, Chen et al. investigated the influence of triazoles antifungal drugs on the pharmacokinetics of zanubrutinib and acalabrutinib, two Bruton’s tyrosine kinase inhibitors commonly used in the therapy of B-cell malignancies. The PBPK models were computed by software and validated using clinically observed plasma concentrations reported in published studies. The authors successfully described the zanubrutinib and acalabrutinib area under the curve (AUC) increase due to the concurrent oral administration of voriconazole, fluconazole, and itraconazole at different dosages. The *in silico* prediction of drug-drug interaction using a PBPK model was performed by Granana-Castillo et al. to assess the possible pharmacokinetic interaction between rifapentine and rilpivirine. Rilpivirine bioavailability variation was observed in both the administration regimens simulated, with a higher decrease of AUC_0-24_ in the simulation of concomitant administration of rifapentine with rilpivirine at the steady state. The obtained results suggested avoiding the coadministration of the two drugs. Furthermore, the olverembatinib drug-drug interaction was investigated by Yu et al. applying a PBPK model to evaluate the possible pharmacokinetic interaction CYP450 mediated. The used model allowed for the successful prediction that CYP3A4 inhibitors may increase olverembatinib plasma concentrations, while the CYP3A4 inducers may decrease its AUC. Conversely, the impact on other CYP450 isoforms was negligible.

Considering the importance of single CYP450 isoforms in drug metabolism, the polymorphism of such enzymes may play a crucial role. To this concern, Ye et al. investigated the influence of CYP2D6 variations on fluvoxamine pharmacokinetics and the drug-drug interaction with apatinib in an *in vivo* model, using Sprague Dawley rats. The study showed that apatinib inhibits fluvoxamine metabolism according to a mixed mechanism and the gene polymorphisms can remarkably affect plasma availability. Similarly, the genetic variability of CYP3A4 may affect pyrotinib pharmacokinetics as elucidated by Zhang et al. The authors applied a PKPB model to evaluate the potential risk of pyrotinib coadministration with strong inhibitors and to quantitatively estimate potential drug-drug interaction for CYP3A4 modulators. Moreover, ketoconazole, fluconazole, and itraconazole may reduce the clearance of pyrotinib *in vitro* and *in vivo* model, as reported by Wang et al.

The growing interest in natural active principles pushed the scientific community to investigate the possible drug-drug interactions with the most used pharmaceutical drugs. Differently from synthetic pharmaceutical drugs, natural remedies from traditional medicine are composed of different active principles, which produce therapeutic effects acting in synergy. The pharmacokinetic herb-drug interactions (HDI) between *Polygonum capitatum* Buch.-Ham. ex D. Don extract and ciprofloxacin were investigated by Li et al., according to a randomized, three-period, crossover trial in healthy humans. Furthermore, the tissue distribution was evaluated in rats. Multiple transporter-mediated HDI contributes to the effects of *P. capitatum* on the reduced systemic exposure and altered tissue distribution of ciprofloxacin. The natural active principle *Epimedium sagittatum* interaction with CYP3A4 was investigated by Li et al. to evaluate the possible drug-drug interaction with corticosteroids. Epimedium resulted to inhibit CYP3A4 activity according to a dose-dependent mechanism in the HepG2 cells model, enhancing the corticosteroids’ anti-inflammatory effect.

The molecular mechanism of action of the natural active principle Ginkgolide B was the focus of Cao et al., who studied the inhibition function of Ginkgolide B on neuronal apoptosis after cerebral ischemia in an *in vivo* rat model and *in vitro* cultured SH-SYS5Y cells model. The results suggest that the natural active principle may reduce neuronal apoptosis preventing ischemic stroke. An innovative computational approach was applied by Yuan et al. to construct a protein-protein interaction network in order to study the effect of Xuefu Zhuyu Decoction in the treatment of atherosclerosis**.** A similar computational approach was applied by Zhou et al. to construct molecular subtypes of colon cancer and subsequently explore prognostic genes with GEPIA2. The machine learning-based study allowed proposing new colon cancer prognostic markers.

Analytical methods represent an important aspect of drug-drug interaction pharmacokinetic studies. To this concern, Tang et al. developed and validated a UHPLC-MS/MS method to quantify almonertinib in rat plasma. The method was successfully applied to study the pharmacokinetic interaction between paxlovid and almonertinib in an *in vivo* rat model.

Finally, drug-drug interaction studies may elucidate important aspects of polytherapy-induced drug resistance. Ghosh et al. examined the interactions of antiseizure drugs in isolated brain cells from patients with drug-resistant epilepsy, highlighting that antiseizure drugs modulate pro- and anti-apoptotic protein levels *via* a CYP-mediated mechanism.

In conclusion, this Research Topic is providing updated studies concerning drug-drug interactions between new drugs, natural drugs, and polydrug treatments, proposing new promising computational approaches.
